# Unveiling the deformation and crack mechanism of glass nanostructure embossing: A molecular dynamics study at experimental scale

**DOI:** 10.1371/journal.pone.0344907

**Published:** 2026-03-24

**Authors:** Xueguang Cui, Ping He, Yingjie Xu, Hao Huang, Wuyi Ming, Weiwei Zhang

**Affiliations:** 1 Fair Friend Institute of Intelligent Manufacturing, Hangzhou Vocational & Technical College, Zhejiang, China; 2 Zhe Jiang Ming Bo Auto Parts Co., Ltd, Zhejiang, China; 3 Henan Key Lab of Intelligent Manufacturing of Mechanical Equipment, Mechanical and Electrical Engineering Institute, Zhengzhou University of Light Industry, Zhengzhou, China; 4 Guangdong Provincial Key Laboratory of Digital Manufacturing Equipment&Guangdong Huazhong University of Science and Technology Industrial Technology Research Institute, Huazhong University of Science and Technology, Dongguan, China; 5 Institute of High Energy Physics, CAS, Shijingshan, Beijing, China; IGDTUW: Indira Gandhi Delhi Technical University for Women, INDIA

## Abstract

Glass nanostructure embossing is a critical manufacturing process for producing high-precision glass components used in optics and electronics. However, controlling the deformation and fracture mechanisms of glass during embossing remains a significant challenge due to its complex behavior, which can vary between solid and liquid-like states under different conditions. To investigate these mechanisms, this study employs large-scale molecular dynamics (MD) simulations that mirror experimental conditions. The simulations reveal how compressive forces near the mold interface lead to densification and lateral flow of the glass, while tensile stresses at the edges can promote crack formation. Additionally, the study examines the role of strain rate in crack propagation, showing that higher strain rates accelerate failure. These findings offer a deeper understanding of the atomic-level behavior of glass during embossing, highlighting key factors such as stress distribution, energy evolution, and material flow. By bridging the gap between molecular simulations and experimental observations, this work provides valuable insights into optimizing embossing conditions. The results can be applied to improve the quality of glass nanostructures, reducing defects and ensuring the mechanical robustness of glass-based devices.

## 1. Introduction

**Fig 1:**
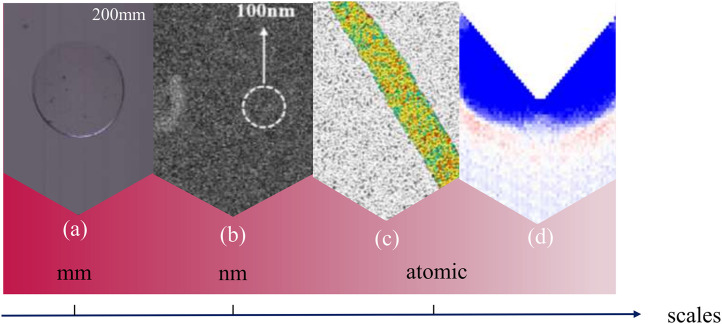
Glass embossing deformation research review at the macroscopic level and molecular dynamics. **(a)** Photograph of dimples produced by continuous laser irradiation of glass [12]; **(b)** SEM image of embossing on 100 nm glass [14]; **(c)** Plastic deformation at a sample size of 100 nm [15]; and **(d)** EDGE stress evolution and stress evolution by displacement. Silicon atoms are coloured by stress and carbon atoms are grey. Oxygen atoms are empty [26]; **(e)** Indentation depth at an indenter angle of 90° [27].

Glass embossing is an advanced manufacturing process that applies precise pressure to the surface of a glass material in order to create complex three-dimensional patterns on it [1]. This technology provides critical material support for several high-tech fields through an efficient and cost-effective process. As shown in Fig 1, in the field of optics, its microstructure array design can effectively reduce the reflection of light and improve the transmittance and efficiency of optical devices, significantly improving the performance of optical products [2]. In electronics, nano structure glass is characterized by its low dielectric loss characteristics and the ability to improve heat dissipation, providing a solid and high performance material [3]. In addition, in microelectromechanical systems (MEMS), the functionality of glass has been further enriched through the embedding of nanoscale patterns [4], which have been applied in the fabrication of key components such as MEMS sensors, actuators, and microfluidic devices [5].

Glass embossing shows a wide range of applications and significant practical value, and this field attracts numerous scholars to conduct in-depth investigation of hot embossing technology. Specifically, in the improvement of embossing technology, Li et al. [6] developed a rapid embossing equipment, which is suitable for creating fine microstructures on high softening point glass by increasing the embossing force, embossing temperature and soaking time, and slowing down the annealing speed, which effectively enhances the filling properties of glass materials. As for the numerical approach, Yun et al. [7] used the finite element method (FEM) combined with the arbitrary Lagrangian-Eulerian (ALE) re-gridding technique to analyze the physical phenomena during the embossing process. As for process optimization, Haponow et al. [8] successfully fabricated and optimized the inverted morphology of the micro-pyramidal cavity structure on top of a thermoplastic sheet by roll-to-roll embossing technique.

However, researchers still face significant challenges in understanding and precisely controlling the mechanics of glass microstructural deformation and its crack initiation during embossing. The central source of these challenges lies in the amorphous structure of the glass, which lacks a well-defined crystalline ordering [9], resulting in a disordered state of the glass at the atomic level. This disorder makes it difficult to accurately predict the mechanical response of the glass, as the glass may exhibit both solid and liquid properties under different conditions [10]. In addition, the brittle nature of the glass, and the size of the microstructure further significantly reduce the toughness, making the glass microstructure subject to instantaneous fracture potentially leading to catastrophic structural failure, thus severely limiting the structural integrity and service life of nanostructured glasses [11], and such instantaneous phenomena are difficult to observe experimentally, and therefore the mechanism of fracture is difficult to investigate.

There have been several studies focusing on the mechanistic principles of glass deformation and cracking, and exploring the macroscopic behavior of glass under various loading conditions, but these studies have often been limited to large scales. Quantitative analysis of glass deformation at the nanoscale is crucial in acquiring a deeper understanding of the mechanical response during the embossing process. Fig 1 provides a brief review of the current research on nanostructure at different size scale. Kurita et al. [12] provided a novel processing method through a laser-heated glass embossing process, but from the beginning to the end of the process, the mold displacement increased by 0.5 μm, as shown in Fig 1a, and it was shown that the change in the displacement was independent of the depth of the process and came from the deformation of the glass. Zhou et al. [13] investigated precision glass forming techniques to develop mold fabrication methods for millimeter scale and high-quality micro- and nanostructures to meet the functional requirements of micro- and nanostructured glass devices And later on, Li et al. [14] found that with 100 nm of the nanostructure of the embossed glass exhibited nanoscale effects during the extrusion flow, which can be seen in Fig 1b, providing initial insights into the understanding of the embossing mechanism. In addition, Julia R. Greer et al. [15] investigated the plastic deformation of glass at different nanoscale scales, as shown in Fig 1c, experiencing a transition from localized to homogeneous deformation as the size of the sample was reduced to 100 nm. Zhao et al. [16] make an important contribution by combining double nanoscratch experiments and MD simulations to clarify how loading force, scratch spacing, and crystallographic orientation govern atomic-scale removal and subsurface damage in GaN. Zhao et al. [17] significantly advance understanding of fused silica machinability by linking double-scratch spacing to the evolution of various crack types, material removal rate, and surface roughness through carefully constructed MD models and FIB–TEM characterization. Zhao et al. [18] provide valuable insight into ultra-precision machining of single-crystal MgAl₂O₄ by revealing how different multiple-scratch sequences affect deformation and removal mechanisms and by proposing penetration-depth models that agree closely with experimental observations.

However, these studies remain qualitative and the precise atomic scale mechanisms of deformation and crack extension during embossing remain unclear, and quantitative molecular level analyses of glass deformation during nanoscale embossing are needed to fill this knowledge gap. Molecular dynamics (MD) simulations provide a powerful method for quantitatively investigating the mechanical behavior of glasses at the nanoscale. MD simulations allow detailed observation of atomic interactions during deformation, which can capture crack initiation, stress distribution and material flow dynamics with high accuracy [19]. Ma et al. [20] incorporated Voronoi’s polyhedral theory and constructed an icosahedral cluster model to describe the structural characteristics of glass, in which the icosahedral structure has perfect fivefold symmetry [21]. The results of MD simulations further revealed the relationship between the local fivefold symmetry and physical phenomena, such as the mechanism of plastic deformation and the glass transition in metallic glasses [22]. And later Murali et al. [23] recognized from the atomic scale through molecular dynamics simulations that the fracture of brittle metallic glasses results from an inherent cavity mechanism dominating the crack extension and that this cavity behavior originates from spatial fluctuations in the atomic scale of the metallic glass microstructure. Although MD is effective in understanding nanoscale processes, most studies are still limited by computational resources to replicate the scale of real-world experimental environments [24,25]. As a tool for quantitative analysis of microscale variations, our team developed a specific MD model to simulate the glass molding process and the subsequent rebound effect, which provides an atomic-level view of the displacement variations during the demoulding process, deepens the knowledge of the rebound mechanism at the atomic level, and points out that the EDGE component is subjected to maximum pressure due to the minimization of the area perpendicular to the direction of the compressive force, which in turn undergoes significant plastic deformation, as shown in Fig 1d [26]. In addition, Liu et al. [27] implemented a 3D nanoindentation study through MD simulation, focusing on the deformation and cracking response of glass materials under sharp contact loads, and quantitatively analyzed the effect of indenter angle on the nanoindentation characteristics, and found that when the indenter angle was 90°, the indentation depth reached a maximum value of 37.5 nm as shown in Fig 1e, which lays a theoretical and practical foundation for the design of glass materials with damage-resistant properties. This lays a theoretical and practical foundation for the design of glass materials with damage-resistant properties. However, the high computational cost of MD simulation limits the scale of simulation, which makes it difficult to directly correspond to the macroscopic experimental results, and the influence of the size effect, the conclusions obtained by MD are still limited in revealing the experimental mechanism.

In this study, we present a large-scale molecular dynamics simulation matching the size of the experimental glass embossing. The deformation and cracking mechanisms of glass nanostructured embossing are revealed through detailed MD analyses, providing insights into how strain, stress, and crack formation evolve during the embossing process. Also, combining our MD model with experimental conditions provides a comprehensive understanding of the material response, which is essential for optimizing the embossing technique to produce defect-free glass nanostructures. This study helps to bridge the gap between molecular modeling and experimental observations, providing new perspectives on the mechanics of glass deformation and damage. These findings are critical for increasing the reliability of glass nanostructure fabrication processes, improving defect control, and ensuring the mechanical robustness of glass-based devices at the nanoscale.

## 2. Modeling

• Geometry

**Fig 2:**
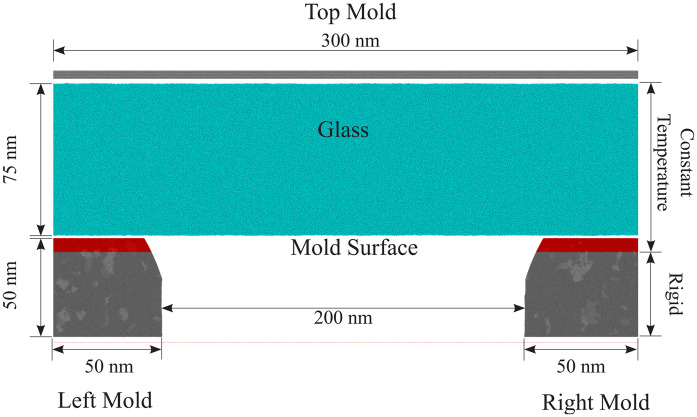
System setup region. Atoms are colored by different control methods and types. The gray and red atoms are Cr atoms, and the former ones are set rigid, and the latter ones are under constant temperature control. The blue atoms consisting of Si and O atoms are also under constant temperature control.

The width of the cavity in the glass embossing experiment is 200 nm, and the maximum depth of the glass after embossing is about 23 nm [28]. We established the simulation system with the identical dimensions of the experiment, as shown in Fig 2. The width is 200 nm, and the depth of the cavity is 50 nm, which is larger than the maximum height of the glass after embossing. It is crucial to note that the thickness of the simulated glass (75 nm) is sufficiently large for the deformation process to occur away from the lower interface, where boundary effects could potentially distort the results. The thickness is more than three times the experimental penetration depth (≈23 nm) and 1.5 times the cavity depth, ensuring that the active deformation region is well removed from the bottom interface. This thickness is adequate for studying the embossing mechanism and forming the desired nanostructures, as the deformation and densification primarily occur near the top mold–glass interface. Additionally, periodic boundary conditions in the lateral direction further minimize the potential for lateral confinement effects. Periodic boundary conditions(PBC) are applied in the horizontal direction to simulate parallel cavities as in the experiment, which are a technique used to model a small, repeating portion of a larger system by simulating the interactions between particles in a finite volume. Instead of simulating a single isolated system, PBCs make the system behave as if it were infinitely extended in space.. The width of the mold is 100 nm, which is large enough to avoid the interaction between cavities. The angle is assumed to be 60°. Although the thickness of the glass in the simulation is 75 nm, which is far thinner than the glass in the experiment, it is thick enough to form the nanostructures in the experiment. The top mold is 3 nm thick.

- Potential Function and ensembles

The potential function defines the interaction between atoms, which is crucial for the accuracy of the MD simulation. We implement the 2NNMEAM + Qeq interatomic potential. The 2NNMEAM + Qeq potential, combining the second nearest-neighbor modified embedded-atom method (2NNMEAM) with the charge equilibration (Qeq) scheme, offers a significant advantage in modeling materials with complex bonding characteristics, such as metallic, covalent, and ionic interactions. Previous studies have shown that this potential provides a more accurate representation of multicomponent systems like metal-oxide composites, which are particularly relevant to the behavior of glass materials under deformation, as studied in our paper. The choice of this potential was driven by its ability to simultaneously capture the key bonding interactions in materials like glass, which exhibit metallic, covalent, and ionic bonding. Additionally, the Qeq method allows for charge equilibration, addressing charge transfer between atoms during deformation, which is essential in understanding the mechanical behavior of complex materials at the atomic scale. This makes the 2NNMEAM + Qeq potential particularly suitable for our study of glass deformation and crack formation, as we aim to capture both the material’s structural integrity and the intricate atomic-level behavior under stress.

This potential addresses limitations in existing models that often specialize in only one or two bonding types, enabling simulations of complex multicomponent systems like metal oxides, ceramics, and composites [29]. The potential function can well reproduce the physical properties of glass and proves to be accurate in many research [30,31].

In molecular dynamics (MD) simulations, an ensemble refers to a statistical framework that defines the thermodynamic conditions under which a system evolves. Common ensembles include: NVE (microcanonical ensemble): The system has a constant number of particles (N), volume (V), and energy (E). It is used to simulate isolated systems with no energy exchange with the environment. NVT (canonical ensemble): The system has a constant number of particles (N), volume (V), and temperature (T). It is used to simulate systems in thermal equilibrium with a heat bath. NPT (isothermal-isobaric ensemble): The system has a constant number of particles (N), pressure (P), and temperature (T). It is used to simulate systems under controlled pressure and temperature, allowing volume fluctuations.

In our MD simulations, we first use the NPT ensemble to relax the system to its equilibrium state. This involves adjusting the system’s volume and temperature to reach a stable configuration that matches the desired pressure and temperature conditions. Once the system is equilibrated, our MD simulation switch to the NVT ensemble to study the system’s dynamics under constant temperature conditions.

- Temperature setting and model preparation

The glass transition temperature (T_g_) is a critical parameter in understanding the thermal behavior of amorphous materials like glasses and polymers. It represents the temperature at which a material transitions from a hard, glassy state to a softer, rubbery state. One effective way to determine T_g_ is by analyzing how the material’s density changes with temperature [32]. In the glassy state, the material’s molecules are in a frozen, disordered arrangement. As the temperature increases, the density decreases slightly due to thermal expansion, but the rate of change is relatively low. Once the temperature surpasses T_g_, the molecular chains gain increased mobility. This leads to a more significant decrease in density with temperature because the material expands more readily.

**Fig 3:**
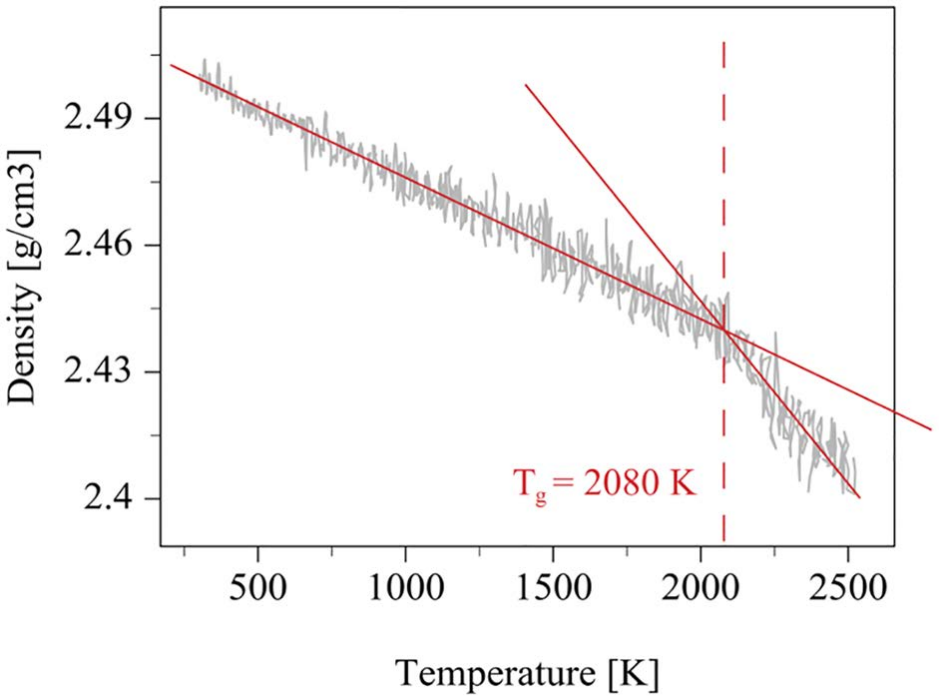
Temperature dependent density and glass transition temperature.

To determine the glass transition temperature of the current potential energy in MD simulation, we heat a cubic glass system and plot the relationship between density and temperature as shown in Fig 3. And, we estimate the T_g_ is about 2080 K.

In the experiment [28], the glass transition temperature T_g_ of the D-K9 glass is 769 K. The glass was heated to 803 K (=1.044 T_g_) before embossing, and the embossing temperature was 823 K(=1.07 T_g_). Since the glass transition temperature of the glass predicted by the potential function is 2080 K, the glass in the MD simulation is prepared at 2171.52 K(=1.044 T_g_) after fully melting at 5000 K under constant pressure control at 0 GPa for 200 ps. The embossing temperature during simulation is kept constant at 2225.6 K. The top Cr mold is prepared at 2171.52 K under constant pressure and split into left mold and right mold. It is not necessary to split Cr mold, since periodic boundary conditions are implemented. But to visualize the embossing processing better, we split the top molds into left and right. Besides splitting, we also cut the corners of the molds since ultra-sharp corners are impossible to yield.

The simulations were conducted using LAMMPS (Large-scale Atomic/Molecular Massively Parallel Simulator) with a timestep of 2e-3 femtoseconds. The NVT ensemble (constant number of particles, volume, and temperature) was used to control the temperature, while the Langevin thermostat was employed for the boundary regions. The cooling rate was set by the variable timestep $(100.0*dt) to accurately determine the glass transition temperature (Tg), which was found to be approximately 2080 K after cooling from 5000 K. Periodic boundary conditions were applied in the horizontal direction to simulate an infinite system, replicating the computational box during the embossing process.

- Embossing setup

The temperature during embossing is kept constant at 1512 K(=1.07 T_g_). The mold is moving at constant speed at 4 angstrom/ 500 fs = 8 angstroms/ps, and keeps rigid during the cooling stage. the mold speed used in the simulation, which is 8 Å/ps, corresponding to a mold velocity of approximately 800 m/s. This speed is significantly higher than typical experimental speeds. As a result, the strain rates in the simulation are naturally higher than those encountered in real-world experiments.

However, it is important to clarify that the high mold speed does not invalidate the overall findings of the simulation. While the strain rate in the simulation is faster than in the experimental setup, the material’s behavior is primarily governed by its viscosity, especially near the glass transition temperature (Tg). At 1.07Tg, the glass is in the viscous flow regime, where the material behaves in a more fluid-like manner than in its glassy state. The dominance of viscosity ensures that the material undergoes plastic deformation rather than experiencing elastic behavior or shock-like effects, even at the higher strain rates used in our simulations.

The choice of high mold speed was made to complete the simulation within a reasonable timescale while still capturing the essential physics of the embossing process. The material’s response is determined by its viscosity at this temperature, and as such, the higher strain rate resulting from the faster mold speed does not lead to unrealistic deformation or material failure. The stress, potential energy, and density fields evolve smoothly throughout the embossing process, indicating that the material behaves consistently with viscous flow under the applied pressures, without exhibiting any shock compression or elastic deformation. The embossing experiment is performed under constant temperature. In the simulation, the whole system is divided into three regions under various control. The top molds consist of two different regions, namely rigid region and region under constant temperature.

## 3. Results

### 3.1. Atomistic mechanism of embossing

**Fig 4:**
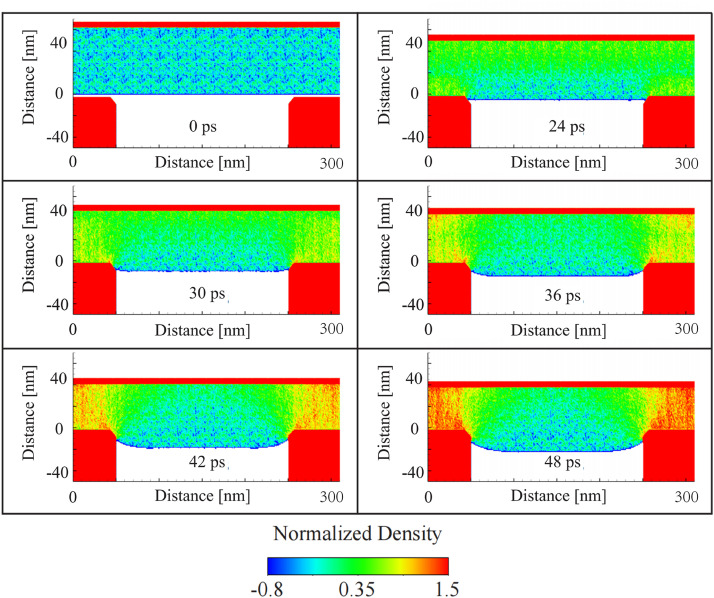
Density evolution during the glass embossing process. The density is normalized to the density of the glass.

The results depicted in Fig 4 illustrate the evolution of normalized density within the glass during the embossing process. The glass is represented by the central cubic region, which is sandwiched between two molds—the top mold is actively pressing down on the glass, while the bottom mold remains rigid. The density in the glass is normalized to the initial density of the unembossed glass, and the progression of the embossing process is shown at various time intervals, spanning from 0 picoseconds (ps) to 48 ps.

At 0 ps, before the initiation of embossing, the glass is undeformed, and its density remains uniform. The molds, shown in red, are positioned above and below the glass, ready to initiate the embossing process. As the process begins and the mold starts to descend, the glass material deforms under the applied pressure. By 24 ps, the density near the top interface of the glass, where it is in contact with the descending mold, begins to increase. This increase in density, indicated by the transition to green and yellow shades in the density profile, can be attributed to the compression of the glass as it flows to fill the mold cavity. The central region of the glass still retains its original density (blue), while the areas closest to the mold experience higher compression and corresponding density increases.

During 30–42 ps, the density distribution becomes more heterogeneous, with high-density regions (yellow and red) forming near the top and low-density regions near the sidewalls of the glass. This distribution indicates that the glass is undergoing significant flow, with material being pushed outward and downward as the mold further penetrates. The bottom mold, remaining rigid, prevents downward displacement, causing the glass to flow laterally. The densification near the contact interface is crucial for ensuring that the glass fills the finer details of the mold pattern, a key objective in the nanostructure embossing process.

At 48 ps, the densification becomes even more pronounced, especially in the central region directly beneath the mold, where the glass is being compacted into the cavity. The bottom mold’s rigidity ensures that the glass is confined, forcing the material to flow into any remaining open space in the mold. The normalized density near the contact areas reaches values significantly higher than 1.0, suggesting substantial local compression, while the surrounding areas exhibit reduced density (blue to green), indicating material displacement away from the compression zone.

The density evolution during the glass embossing process provides valuable insights into the material’s response to applied pressure and temperature. The formation of high-density regions near the mold interface indicates effective compression and material flow, while the lateral displacement and lower-density regions highlight areas where optimization may be required. These findings are crucial for refining the embossing process to achieve precise, defect-free nanostructures in glass materials.

**Fig 5:**
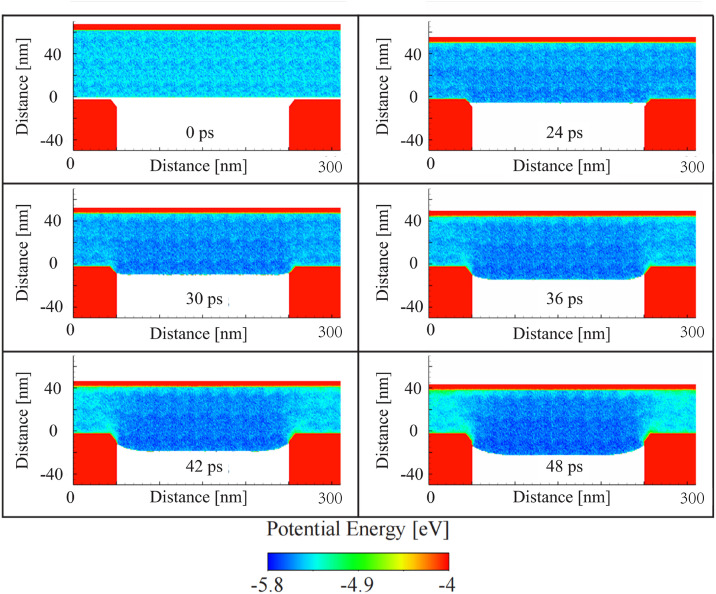
Potential energy evolution during the glass embossing process.

The results presented in Fig 5 illustrate the evolution of potential energy in the glass material during the embossing process, as a function of time. The potential energy values are depicted using a color gradient from −5.8 eV to −4 eV.

At 0 ps, before the embossing process begins, the potential energy is low (approximately −5.8 eV), reflecting the stable structure of the glass before any applied external forces. At this stage, the molds are in position but have not yet made contact with the glass surface. From 24 ps to 36 ps, as the top mold begins to move and apply pressure to the glass, changes in potential energy become noticeable. The area directly beneath the top mold begins to show a slight increase in potential energy, transitioning from darker blue to a lighter blue-green. This shift suggests that the applied pressure is causing localized deformation in the glass, resulting in an increase in atomic interactions and potential energy in the compressed regions. The central region of the glass, however, remains largely unchanged, indicating that the deformation has not yet propagated fully through the glass structure.

By 42 ps and 48 ps, the potential energy distribution stabilizes somewhat, though regions of elevated potential energy remain visible near the interface between the top mold and the glass. This suggests that the material has largely accommodated the imposed deformation but continues to experience stress near the mold contact areas. The bottom region of the glass, next to the rigid mold, shows relatively lower potential energy, showing that this part of the glass is not as significantly affected by the embossing force and remains more structurally intact. However, the overall energy distribution indicates that the glass has reached a deformed state with some localized regions still experiencing higher energy due to the ongoing interactions with the molds.

The evolution of potential energy during the embossing process provides a detailed view of the atomic-level changes occurring in the glass as it undergoes deformation. The increase in potential energy near the mold interface reflects the material’s response to mechanical forces, while the stabilization of potential energy over time suggests that the glass reaches a deformed, yet stable, configuration. These insights are critical for optimizing embossing conditions to achieve defect-free nanostructures with uniform material properties.

**Fig 6:**
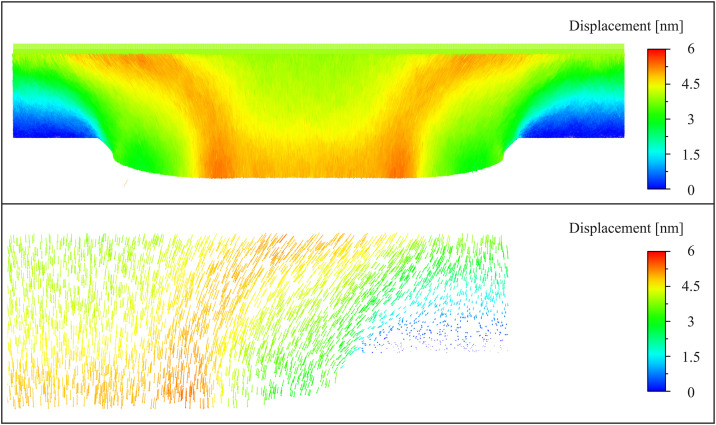
Displacement vectors between 42 ps and 48 ps. The bottom figure is the zoomed-in view of the top figure on the right. The displacement vectors are chosen randomly to better visualize the arrows.

Fig 6 illustrates the temporal evolution of stress within the glass material during the embossing process. The stress is color-coded: tensile stress is shown in red and yellow, while compressive stress is indicated in blue. The figures show the stress distribution at various time intervals as the mold applies pressure to the glass surface.

At 0 ps, before any deformation occurs, the glass is in a relaxed state with negligible internal stresses. As the mold begins to descend, 24 ps shows the initial buildup of compressive stress at the mold-glass interface. This stress increase results from the applied pressure, which forces the material into the mold. The transition from blue to green at the interface indicates the development of compressive stresses, which are expected as the glass begins to undergo densification.

By 30 ps, the stress distribution becomes more heterogeneous, with tensile stresses developing in the lateral regions of the glass. The mold’s pressure causes the glass to flow laterally, leading to stretching and the formation of tensile regions away from the point of direct contact. The stress in these lateral regions reaches a maximum at 42 ps, indicating the propagation of strain in the material as it flows to accommodate the mold’s shape.

At 48 ps, the glass material has largely conformed to the mold’s shape, and the stress profile begins to stabilize. However, regions near the mold edges still exhibit elevated tensile stresses, suggesting that the material continues to undergo small adjustments as it reaches an equilibrium state. These tensile zones are of particular interest, as they represent areas where fracture initiation is more likely to occur due to the concentration of stress.

The stress evolution during the embossing process provides valuable insights into the material’s response to the applied forces. The development of compressive stress at the mold-glass interface is expected, as this is the region most directly affected by the mold’s pressure. The lateral flow of the glass material, as indicated by the tensile stress buildup, underscores the importance of understanding the material’s viscoelastic properties, particularly near the glass transition temperature (Tg), where the material behaves in a more fluid-like manner.

The persistence of tensile stresses at the mold edges even after the main deformation occurs is a critical observation. These regions of high tensile stress are more prone to fracture, as the material’s resistance to crack propagation is lower in tensile zones compared to compressive zones. The risk of fracture in these areas is compounded by the relatively low toughness of glass in its amorphous state under high strain rates, a behavior that is commonly observed in the context of brittle materials subjected to high rates of deformation.

Fig 6 presents the displacement field of the glass material between 42 ps and 48 ps during the embossing process. The displacement is shown in two parts: the top figure illustrates the overall displacement field across the entire embossing region, while the bottom figure provides a zoomed-in view on the right-hand side for better visualization of the vector directions and magnitudes. The displacement vectors have been randomly selected to facilitate the clear representation of movement patterns, with colors corresponding to displacement magnitudes, ranging from 0 nm (blue) to 6 nm (red).

In the top portion of Fig 6, the displacement magnitudes are highest near the center of the contact region between the mold and the glass (in red and yellow), where the mold exerts the greatest pressure. As the glass deforms under the compressive force from the mold, it is forced both downward and laterally outward. The color gradient indicates a high displacement magnitude (up to 6 nm) in the central region of the glass, while the displacement decreases (towards blue) as we move farther from the mold contact region. The material near the lateral edges of the mold, which has already been subjected to displacement earlier in the embossing process, shows comparatively lower displacement magnitudes. This suggests that most of the material deformation is now concentrated near the center, where the mold continues to press downwards.

In the bottom portion of Fig 6, a more detailed view of the right-hand side displacement field is provided. This zoomed-in section offers a clearer depiction of the vector orientations, highlighting the material flow direction. The displacement vectors show a combination of downward and outward lateral movement, confirming that the glass is being pushed not only directly downward by the mold but also radially outward toward the sides. The arrows are directed away from the center of the compression zone, indicating that the material is spreading to accommodate the embossing process. The magnitude of displacement decreases in the areas farthest from the mold, suggesting a diffusion of stress and a gradual tapering of material flow as the glass moves toward a more stable configuration.

The displacement field results shown in Fig 6 provide critical insights into the mechanical response of the glass during the final stages of the embossing process. The large displacements observed in the central region beneath the mold indicate that the material experiences significant deformation in response to the applied compressive load. This behavior is expected, as the glass is softened at 1512 K (1.07 Tg), allowing for viscous flow under the applied pressure. The fact that displacement is highest in the central region and decreases outward suggests that material flow is concentrated near the primary point of contact between the mold and the glass, while the regions farther from the mold are less impacted by the ongoing deformation.

The embossing mechanisms of glass nanostructures, as revealed by the analysis of the provided figures, can be summarized as a dynamic interaction between mechanical pressure, material deformation, and the redistribution of stress, energy, and displacement. Throughout the process, the glass undergoes significant changes in density, potential energy, stress, and displacement, all of which contribute to the formation of the embossed nanostructure.

In conclusion, the embossing mechanism of glass nanostructures involves a complex interplay of compressive forces, material flow, and atomic-level structural changes. The material deforms under the applied pressure, with densification and potential energy increases localized near the mold-glass interface. Stress gradients and displacement patterns reflect the redistribution of glass as it flows into the mold, with regions of high stress and displacement corresponding to areas of significant material deformation. These results highlight the importance of optimizing the embossing conditions, including pressure, temperature, and mold speed, to achieve uniform filling and defect-free nanostructures.

**Fig 7:**
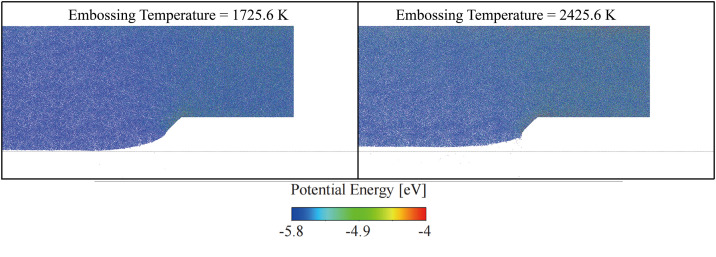
Temperature effect on embossing at 40 ps.

Fig 7 illustrates the impact of embossing temperature on nanostructures by showing the potential energy distribution at different temperatures (1725.6 K vs. 2425.6 K). At the lower embossing temperature of 1725.6 K, the structure retains more localized energy variations, indicating that the material remains relatively rigid and less able to flow and conform to the mold. As a result, the nanostructure may suffer from incomplete filling or sharp edges with higher potential for defects. In contrast, at the higher temperature of 2425.6 K, the material exhibits more uniform energy distribution, reflecting increased fluidity and ease of filling. This enables the nanostructure to better conform to the mold, likely resulting in smoother, more continuous features with less risk of sharp edges or voids. However, the higher temperature also raises the possibility of material flow beyond desired areas or potential shape distortion if not carefully controlled.

### 3.2. Crack mechanism of glass nanostructure

The crack mechanism in glass nanostructures is vital to their mechanical reliability, defect control, and long-term performance. Using MD simulations to investigate crack mechanisms provides atomic-scale insights, predictive capabilities, and the ability to simulate extreme and nanoscale phenomena, all of which are critical for developing optimized processes and more resilient glass nanostructures. By studying crack mechanisms through MD, we can help refine the design and manufacturing of *glass* nanostructures to reduce defects, improve strength, and enhance the durability of glass-based devices.

**Fig 8:**
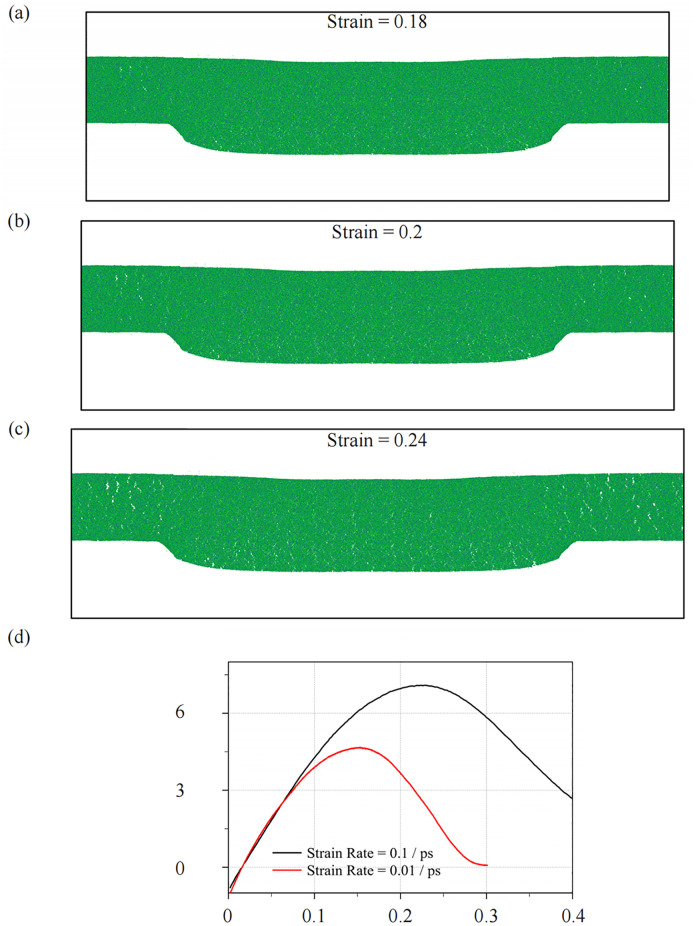
Crack evolution during MD tensile test and stress-strain curve under different strain rates.

Fig 8 illustrates the evolution of crack formation during the molecular dynamics (MD) tensile test on the glass material, with corresponding stress-strain curves at different strain rates. The crack initiation, propagation, and the stress-strain relationship are depicted at atomic scales, providing an in-depth view of how microcracks evolve into macroscopic fractures under tensile loading conditions.

At the atomic level, crack initiation begins when the tensile stress applied to the glass reaches localized regions of stress concentration. These areas are typically near the central part of the specimen, as shown in Fig 8(a) (strain = 0.18). At this early stage, atomic rearrangements occur as the glass responds to the increasing stress. The atoms in the material undergo slight displacements, and small voids begin to form. These voids act as nuclei for crack initiation, a well-documented phenomenon in brittle materials. The local stress distribution, influenced by atomic interactions, reaches a critical threshold that facilitates the formation of these atomic-scale microcracks.

As strain increases (to 0.20 and 0.24 as shown in Fig 8(b) and 8(c)), the microcracks begin to extend and coalesce. The MD simulations reveal that the crack growth is governed by the atomic-level interactions between the silicon (Si) and oxygen (O) atoms in the glass structure. The formation of these cracks is highly dependent on the bond strength between these atoms and their ability to withstand tensile forces. When the bond strength is exceeded, the atomic bonds break, leading to the propagation of cracks.

During this phase, the glass exhibits brittle behavior. Unlike ductile materials, where dislocations can move and deform the material plastically, glass fractures almost immediately once a crack propagates due to the lack of significant atomic-scale plastic deformation. The propagation of cracks follows the path of least resistance, typically along regions where atomic bonds are weaker or where defects, such as voids or inclusions, are present.

The MD simulations allow us to visualize the stress distribution around the crack tip in real time. At the crack tip, the stress concentration is highest, and this is where atomic-scale bond-breaking events occur. The high tensile stress at the crack tip causes rapid crack propagation, as observed in Fig 8(c), where a macro-scale crack begins to form. This rapid propagation is indicative of the glass’s brittle fracture behavior, as the atomic bonds are unable to rearrange themselves to accommodate the stress.

The stress distribution is not uniform across the material; rather, it is highly localized near the crack tip, where atomic interactions are intense. The crack propagation is thus dictated by the strain energy release rate at the crack tip, which is governed by atomic-level processes such as bond rupture and atomic displacement.

Fig 8(d) shows the stress-strain curves at two different strain rates: 0.1/ps and 0.01/ps. At higher strain rates (0.1/ps), the glass exhibits a stiffer response, with a sharp increase in stress, followed by a rapid drop as cracks propagate. This is because the atoms do not have sufficient time to rearrange themselves and dissipate energy through plastic deformation, leading to a more abrupt fracture. The MD simulations reveal that at this faster strain rate, the atomic rearrangements are more constrained, which exacerbates the material’s brittle nature and accelerates the crack propagation.

In contrast, at the lower strain rate (0.01/ps), the stress-strain curve shows a more gradual decrease in stress after reaching the peak. This suggests that the material has more time to undergo atomic rearrangements and localized plastic deformation, slowing down the crack propagation and potentially allowing for more energy dissipation. The atomic-scale differences in displacement and rearrangement during these two loading conditions emphasize the strain-rate sensitivity of glass, where a slower loading rate enables more ductile-like behavior, even though the material remains intrinsically brittle.

The crack mechanism observed in the MD simulations is directly relevant to the embossing process discussed in the manuscript. During the embossing of glass nanostructures, the material undergoes complex deformations under high-pressure conditions. The tensile stresses near the mold interface, where cracks are more likely to form, are critical in determining the final quality of the embossed nanostructures. Our findings from the crack evolution in tensile tests can be extrapolated to the embossing process, where similar stress and strain distributions are expected near the mold-glass interface.

The insights gained from the crack initiation and propagation at the atomic scale can help in refining embossing conditions, such as pressure, temperature, and strain rate, to minimize crack formation and optimize material flow. By understanding the atomic-level mechanisms of crack formation, we can propose strategies to reduce defects and improve the mechanical robustness of glass nanostructures in practical applications.

**Fig 9:**
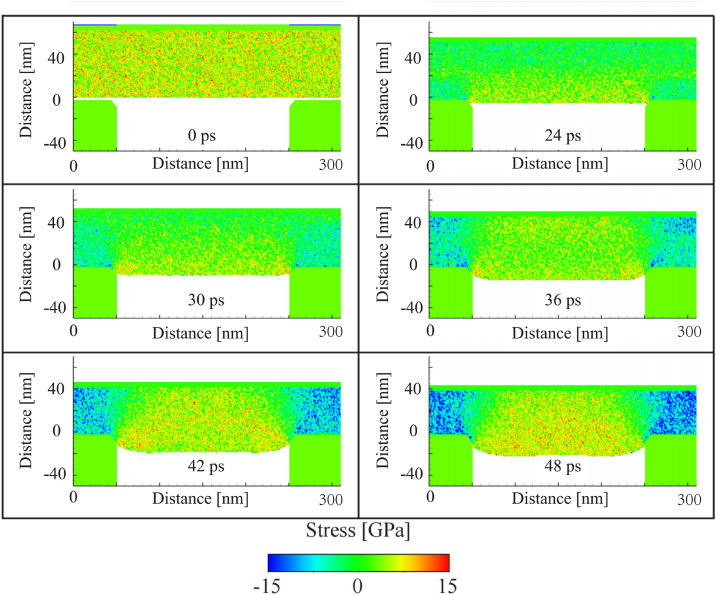
Stress evolution during the glass embossing process.

The strain rate is directly related to the stress and displacement results shown in Figs 6 and 9. As the strain rate increases (as indicated by the black curve in Fig 8), the stress builds up more rapidly in the glass material, which is reflected in the faster progression of compressive and tensile stresses seen in Fig 9. This rapid buildup of stress leads to earlier deformation and more localized material flow, as illustrated by the higher displacement magnitudes in Fig 8. These effects are consistent with the behavior of materials at higher strain rates, where there is less time for atomic rearrangements and more immediate material response to external forces.

Moreover, the relationship between the strain rate and crack formation, as shown in the progression of microcracks in Fig 7, highlights that at higher strain rates, cracks initiate and propagate more quickly. This is reflected in the faster drop in the stress-strain curve at 0.1 ps ⁻ ¹, indicating the material’s earlier failure due to the more abrupt deformation. Conversely, at the lower strain rate of 0.01 ps ⁻ ¹, the crack formation is slower and more gradual, as shown by the red curve in the stress-strain graph. This slower crack propagation allows for more time for atomic rearrangement and energy dissipation, which leads to less catastrophic failure.

The prediction of modulus and tensile strength values for the same material in the same strain rate interval can vary depending on the point model chosen. For example, Ochoa et al. [33] used an optimised Born-Mayer-Huggin (BMH) point model to investigate the effect of strain rate on the properties of amorphous silica and measured strengths ranging from 30 to 65 MPa under uniform deformation conditions. In contrast, Swirler et al. [34] employed the two-body Suress potential model for the same material and obtained a strength value of 344 MPa. In addition, Muralidharan et al. [35] analysed the behaviour of silica under tensile loading using a modified van Beest-Kramer-van Santen (BKS) potential model which measured tensile strengths ranging from 17 to 20.5 MPa. Therefore, when modeling tensile strengths, the values are not only affected by the strain rate but are also closely related to the potential function chosen, the point model, and other factors. Nevertheless, the mechanism of crack formation presents similarities in these studies. Therefore, in the development of the above models, although there are some deviations between the simulated tensile strength values and the experimental data, this paper mainly focuses on embossing and crack formation mechanisms, and such errors do not hinder the core objective.

In conclusion, the MD simulations provide an atomistic-level understanding of crack initiation, propagation, and the effect of strain rates on the mechanical behavior of glass. The crack mechanism in glass nanostructures involves a complex interplay of atomic interactions, stress concentrations, and strain energy release, which are critical for controlling defects in the embossing process. By studying these phenomena at the atomic scale, we gain valuable insights that can be used to optimize manufacturing conditions and enhance the performance of glass-based devices.

## 4. Conclusion

In this study, molecular dynamics (MD) simulations based on the 2NNMEAM + Qeq potential were used to investigate the deformation and cracking mechanisms of glass during nanostructure embossing. This atomistic approach complements macroscopic methods by resolving local stress, energy, and deformation fields at the nanoscale, which are difficult to capture with continuum models. Our main findings can be summarized as follows:

### Embossing mechanism of glass nanostructures

The simulations reveal that embossing is governed by a coupled evolution of density, potential energy, stress, and displacement near the mold–glass interface. Under the applied pressure at 1.07 Tg, the glass undergoes localized densification and viscous flow, with high compressive stress and large displacement concentrated beneath the mold and gradually decaying toward the periphery. These results clarify how material redistribution and stress gradients jointly determine the filling behavior of the nanocavity.

### Strain-rate-dependent deformation and fracture

The stress–strain responses and crack evolution demonstrate a clear strain-rate sensitivity. At higher strain rates, the glass exhibits higher peak stress and more abrupt stress drops, indicating rapid crack initiation and brittle failure due to limited time for atomic rearrangement. At lower strain rates, stress builds more gradually and failure is less catastrophic, suggesting that slower loading can promote more distributed damage and delayed fracture.

### Crack initiation and propagation mechanisms

Cracks nucleate in regions of tensile stress concentration and evolve from microcracks to a dominant macroscopic crack through growth and coalescence. The MD results identify preferred crack paths and critical regions near stress concentrations, providing microscopic insight into how processing conditions and loading histories can trigger premature failure.

Overall, this work shows that MD simulations can bridge experimental observations and macroscopic modeling by supplying atomistic-level mechanisms for embossing and cracking in glass nanostructures. The insights obtained here can be used to guide the selection of embossing pressure, temperature, and loading rate to improve filling uniformity, reduce defect formation, and enhance the mechanical reliability of glass-based micro- and nanostructured devices. Future work will extend this framework to more complex mold geometries and compositional variations to further support process optimization and material design.

## Supporting information

S1 File EmbossingScript. (ZIP)
